# The molecular consequences of FOXF1 missense mutations associated with alveolar capillary dysplasia with misalignment of pulmonary veins

**DOI:** 10.1186/s12929-024-01088-5

**Published:** 2024-11-04

**Authors:** G. G. Edel, M. van Kempen, A. Boerema-de Munck, C. N. Huisman, C. A. P. Naalden, R. W. W. Brouwer, S. Koornneef, W. F. J. van IJcken, R. M. H. Wijnen, R. J. Rottier

**Affiliations:** 1 Department of Pediatric Surgery, Erasmus MC-Sophia, Rotterdam, The Netherlands; 2 Department of Cell Biology, Erasmus MC, Faculty Building, Room Ee-1034B, Wytemaweg 80, 3015 CN Rotterdam, The Netherlands; 3 Erasmus Center for Biomics, Erasmus MC, Rotterdam, The Netherlands

**Keywords:** FOXF1, Alveolar capillary dysplasia with misalignment of the pulmonary veins, ACD/MPV, DNA-binding domain, Missense mutations, Phosphorylation

## Abstract

**Background:**

Alveolar capillary dysplasia with misalignment of pulmonary veins (ACD/MPV) is a fatal congenital lung disorder strongly associated with genomic alterations in the Forkhead box F1 (FOXF1) gene and its regulatory region. However, little is known about how FOXF1 genomic alterations cause ACD/MPV and what molecular mechanisms are affected by these mutations. Therefore, the effect of ACD/MPV patient-specific mutations in the *FOXF1* gene on the molecular function of FOXF1 was studied.

**Methods:**

Epitope-tagged FOXF1 constructs containing one of the ACD/MPV-associated mutations were expressed in mammalian cell lines to study the effect of FOXF1 mutations on protein function. EMSA binding assays and luciferase assays were performed to study the effect on target gene binding and activation. Immunoprecipitation followed by SDS‒PAGE and western blotting were used to study protein‒protein interactions. Protein phosphorylation was studied using phos-tag western blotting.

**Results:**

An overview of the localization of ACD/MPV-associated FOXF1 mutations revealed that the G91-S101 region was frequently mutated. A three-dimensional model of the forkhead DNA-binding domain of FOXF1 showed that the G91-S101 region consists of an α-helix and is predicted to be important for DNA binding. We showed that FOXF1 missense mutations in this region differentially affect the DNA binding of the FOXF1 protein and influence the transcriptional regulation of target genes depending on the location of the mutation. Furthermore, we showed that some of these mutations can affect the FOXF1 protein at the posttranscriptional level, as shown by altered phosphorylation by MST1 and MST2 kinases.

**Conclusion:**

Missense mutations in the coding region of the *FOXF1* gene alter the molecular function of the FOXF1 protein at multiple levels, such as phosphorylation, DNA binding and target gene activation. These results indicate that FOXF1 molecular pathways may be differentially affected in ACD/MPV patients carrying missense mutations in the DNA-binding domain and may explain the phenotypic heterogeneity of ACD/MPV.

**Supplementary Information:**

The online version contains supplementary material available at 10.1186/s12929-024-01088-5.

## Background

Alveolar capillary dysplasia with misalignment of the pulmonary veins (ACD/MPV) is a fatal congenital lung disorder in which most patients develop symptoms within the first 24 h after birth [1]. The lungs of ACD/MPV patients exhibit structural abnormalities, such as misaligned pulmonary veins, a reduced number of pulmonary capillaries and thickening of the alveolar septa [1, 2]. This causes insufficient gas exchange and progressive therapy-resistant pulmonary hypertension in newborns, resulting in a mortality rate of almost 100% within the first month of life [1].

The exact cause of ACD/MPV is not yet known, but it is strongly associated with genomic alterations that involve the *Forkhead box F1 (FOXF1)* gene and upstream regulatory regions [3–8]. Large deletions and more than 50 heterozygous point mutations have been found in the *FOXF1* locus of ACD/MPV patients [1, 3–6]. FOXF1 is a transcription factor of the helix-turn-helix class of proteins with a typical winged helix DNA binding motif and is an important regulator of mouse and human embryonic development [4, 9]. Both *Foxf1* overexpression and *Foxf1* knockout in mice lead to embryonic or perinatal lethality caused by impaired vascular development and lung defects [10–12]. During lung development, FOXF1 is expressed in the lung mesenchyme and is expressed in cell types that originate from the mesenchyme, such as capillary endothelial cells, fibroblasts and peribronchial smooth muscle cells [13–16]. Cell type-specific inactivation of *FOXF1* in mice show dysregulation of several developmental signalling pathways that are important for healthy lung development. For example, *FOXF1*-specific knockout in endothelial cells showed that FOXF1 is involved in vascular endothelial growth factor (VEGF) signalling, and mesenchyme-specific *FOXF1* knockout revealed a role for FOXF1 in canonical Wingless-related integration site (Wnt) signalling [13, 15].

Although *FOXF1* alterations are present in a large proportion of ACD/MPV patients, little is known about the molecular mechanisms through which these alterations cause structural changes in the lung. Previously reported mutations are either point mutations or insertion‒deletion mutations in the FOXF1 protein-encoding sequence, large copy number variations involving the gene itself or its enhancer region [1, 6]. Because most ACD/MPV patients are diagnosed post-mortem, as it is hard to obtain tissue for studying disease onset and progression. Besides, patients exhibit considerable phenotypic differences making it difficult to distinguish the molecular mechanisms that are generally involved in ACD/MPV from patient-specific alterations. Recently, Pradhan et al*.* showed that the FOXF1 S52F mutation causes ACD/MPV in mice and reported reduced STAT3 signalling and activated canonical WNT/β-catenin signalling through *WNT5A* [17, 18]. However, until now this is the only *FOXF1* mutation with a direct mechanistic link to the phenotypic representation of ACD/MPV that is reported. Therefore, we studied the effect of *FOXF1* mutations identified in ACD/MPV patients on the molecular mechanisms of the FOXF1 protein.

The FOXF1 protein has three distinct domains: a forkhead DNA binding domain, a cell type-specific activation domain and a general activation domain [3]. We specifically focused on mutations in the DNA binding domain, which regulates the expression of downstream target genes [3]. We show that FOXF1 proteins with patient-derived mutations in this domain exhibit altered binding to the DNA-binding motifs and/or altered transcriptional activity. In addition, mutations in the DNA-binding domain of FOXF1 resulted in altered phosphorylation of FOXF1. These results indicate that ACD/MPV-related *FOXF1* mutations have diverse effects on FOXF1 protein function and may explain the phenotypic heterogeneity of patients with ACD/MPV.

## Methods

### Cell lines and cell culture

HeLa cells and HEK293T cells were cultured in high-glucose DMEM supplemented with 5% fetal calf serum (FCS) and 1% penicillin/streptomycin (P/S) under standard cell culture conditions. Human endothelial colony forming cells (hECFCs) were cultured in EBM-2 medium supplemented with 10% FCS and 1% P/S together with hydrocortisone, hFGF-B, VEGF, R3-IGF-1, ascorbic acid, GA-1000 and heparin from the EGM-2 Bulletkit (CC-4176, Lonza).

### hECFC isolation and CD31 + flow cytometry analysis

hECFCs were isolated from human lung tissue according to Alphonse et al. with some modifications [19]. Lung tissue was obtained from residual, tumor-free material obtained from lung resection with the approval of the Medical Ethical Committee of the Erasmus MC Rotterdam. Lung tissue was either immediately processed or stored in high-glucose DMEM supplemented with 1% P/S overnight (O.N.) at 4 °C. Chopped lung tissue was digested with 7–10 ml of digestion solution (0.1 U/ml collagenase type I and 0.8 U/mg dispase in DPBS supplemented with CaCl2 and MgCl_2_) per 0.5 g of wet lung weight, and enzymes were quenched with an equal volume of high-glucose DMEM supplemented with 10% FCS and 1% P/S. Approximately 25 µl of CD31-dynabeads was used for up to 1–5*10^9^ cells, and 24 h after isolation, the medium was refreshed, followed by refreshing three times a week until colonies appeared and were ready for further isolation. ECFC colonies were trypsinized with trypsin–EDTA (TE), which was quenched using 10% FCS/PBS and replated for expansion. The cells were purified for another 1–3 rounds with CD31-dynabeads until the cell population was > 95% CD31 + as determined by flow cytometry analysis on a BD LSRFortessa™ Flow Cytometer using a CD31-PeCy7 antibody (1:200, Biolegend) and DAPI (1:10,000, Beckton and Dickinson). After obtaining a pure ECFC population, the cells were either frozen or expanded for further experiments.

### 3D modelling of the FOXF1 DNA binding domain

Three-dimensional modelling of the FOXF1 DNA binding domain of FOXF1 was performed with EasyModeller 4.0 [20]. The protein sequence of the DNA binding domain of human FOXF1 (Supplementary Figure 1) was used as the query sequence, and FOXK2 (PDB ID: 1JXS), FOXO1 (PDB ID: 6qvw), FOXO3 (PDB ID: 2K86), and FOXO4 (PDB ID: 3L2C) were used as templates. Models were visualized using an iCn3D web-based 3D viewer [21].

### Transfection

Cells were transfected using X-tremeGENE HP DNA Transfection Reagent (Roche) according to the manufacturer’s protocol. The ratio of DNA:X-tremeGENE HP DNA Transfection Reagent for HEK293T cells was 1:3 for immunoprecipitation and Phos-tag western blot experiments and 1:2 for other experiments. The ratio was was 1:2 for HeLa cells and 1:3 for HepG2 cells. Transfection complexes were added to cells in high-glucose DMEM supplemented with 1% FCS and 1% P/S for HeLa and HEK293T cells and cells were harvested 24 h after transfection. For HepG2 cells, transfection complexes were added in high-glucose DMEM supplemented 10% FCS 1% NEAA, 1% ultraglutamin and refreshed with complete medium after 24 h and cells were harvested 48 h after transfection.

### Plasmids and cloning

#### FOXF1 mutants

The wild-type FLAG-FOXF1 construct (CloneID OHu23845C) and a modified version of this plasmid lacking the stop codon were purchased from GenScript. The latter was used to construct a C-terminal V5-tagged FOXF1 plasmid by replacing the FLAG tag with a Kozak sequence and an ATG start codon and replacing the stop codon for a V5 tag. The C-terminal FLAG tag was made by replacing the V5-tag with a FLAG-tag at the C-terminus. DNA inserts were ordered as single-stranded oligonucleotides (IDT) and annealed to make double-stranded DNA inserts. Single-stranded oligonucleotide sequences are listed in pJ3M-MST1 (Addgene plasmid: #12203), pJ3MST1 K59R (Addgene plasmid: #12204), pJ3M-MST2 (Addgene plasmid: #12205) and pJ3M-MST2 K56R (Addgene plasmid: #12206) were gifts from Jonathan Chernoff, MD, PhD [22]. MST1 and MST1 K59R were cloned from the pJ3M plasmid into the pcDNA3.1 + plasmid by restriction with HindIII and EcoRI. To clone MST2 and MST2 K56R into pcDNA3.1 + , pJ3M plasmids were digested with SalI and EcoRI, and the pcDNA3.1 + plasmid was digested with HindIII and EcoRI. Blunt ends were created with Klenow, and MST1 and MST2 K56R inserts were ligated into the pcDNA3.1 + vector.

Table 1.

**Table 1:** Oligonucleotide sequences for cloning

Primer name	Sequence (5′-> 3′)
C-terminal FLAG-tag FW	GATCCgattacaaggatgacgacgataagtgaG
C-terminal FLAG-tag RV	AATTCtcacttatcgtcgtcatccttgtaatcG
Kozak FW	AGCTTCGATCGGCCACCATGGGTAC
Kozak RV	CCATGGTGGCCGATCGA
C-terminal V5-tag with stop FW	GATCCGGTAAGCCTATCCCTAACCCTCTCCTCGGTCTCGATTCTACGTGAACCGGTG
C-terminal V5-tag with stop RV	AATTCACCGGTTCACGTAGAATCGAGACCGAGGAGAGGGTTAGGGATAGGCTTACCG

Overhang sequences for ligation are displayed in capital letters

The L56V mutant was incorporated into either wild-type FLAG-FOXF1 of FOXF1-FLAG using the QuickChange Site-Directed Mutagenesis Kit (Agilent Technologies) according to the manufacturer’s instructions with the following modifications to the PCR mix and PCR cycling parameters: for the PCR, 500 ng of dsDNA template was used instead of 5–50 ng, and 10% DMSO was added to the PCR mixture. For PCR cycling, the first step consisted of 1 cycle at 95 °C for 3 min instead of 1 cycle at 95 °C for 30 s. The primers used are listed in Table 2.

**Table 2:** PCR primers used for constructing the L56V mutation

Name	Sequence (5′→3′)
L56V FW	CTATTCCTACATCGCG**G**TCATCGTCATGGCCAT
L56V RV	GATAAGGATGTAGCGC**C**AGTAGCAGTACCGGTA

Missense mutations are bolded and underlined

All other mutants were constructed using GenParts Elite DNA fragments. For each mutation, a DNA fragment was ordered with the BlpI and BsrGI restriction sites, and the digested fragments were exchanged with the wild-type fragment from the FLAG-FOXF1 or FOXF1-FLAG plasmid. The sequences of the DNA fragments are included in Table 3.

**Table 3:** Elite double-stranded DNA fragments used for constructing FOXF1 missense mutants in the F85-S101 region

Mutation	Sequence 5′-> 3′
F85I	AAGCGCCTGAC**GCTGAGC**GAGATCTACCAGTTCCTGCAGAGCCGCTTCCCCTTC**A**TCCGGGGCTCCTACCAGGGCTGGAAGAACTCCGTGCGCCACAACCTCTCGCTCAACGAGTGCTTCATCAAGCTACCCAAGGGCCTTGGGCGGCCCGGCAAGGGCCACTACTGGACCATCGACCCGGCCAGCGAGTTCATGTTCGAGGAGGGCTCCTTTCGGCGGCGGCCGCGCGGCTTCCGAAGGAAATGCCAGGCGCTCAAGCCCA**TGTACA**GCATGATGAAC
G91V	AAGCGCCTGAC**GCTGAGC**GAGATCTACCAGTTCCTGCAGAGCCGCTTCCCCTTCTTCCGGGGCTCCTACCAGG**T**CTGGAAGAACTCCGTGCGCCACAACCTCTCGCTCAACGAGTGCTTCATCAAGCTACCCAAGGGCCTTGGGCGGCCCGGCAAGGGCCACTACTGGACCATCGACCCGGCCAGCGAGTTCATGTTCGAGGAGGGCTCCTTTCGGCGGCGGCCGCGCGGCTTCCGAAGGAAATGCCAGGCGCTCAAGCCCA**TGTACA**GCATGATGAAC
V96L	AAGCGCCTGAC**GCTGAGC**GAGATCTACCAGTTCCTGCAGAGCCGCTTCCCCTTCTTCCGGGGCTCCTACCAGGGCTGGAAGAACTCC**T**TGCGCCACAACCTCTCGCTCAACGAGTGCTTCATCAAGCTACCCAAGGGCCTTGGGCGGCCCGGCAAGGGCCACTACTGGACCATCGACCCGGCCAGCGAGTTCATGTTCGAGGAGGGCTCCTTTCGGCGGCGGCCGCGCGGCTTCCGAAGGAAATGCCAGGCGCTCAAGCCCA**TGTACA**GCATGATGAAC
V96M	AAGCGCCTGAC**GCTGAGC**GAGATCTACCAGTTCCTGCAGAGCCGCTTCCCCTTCTTCCGGGGCTCCTACCAGGGCTGGAAGAACTCC**A**TGCGCCACAACCTCTCGCTCAACGAGTGCTTCATCAAGCTACCCAAGGGCCTTGGGCGGCCCGGCAAGGGCCACTACTGGACCATCGACCCGGCCAGCGAGTTCATGTTCGAGGAGGGCTCCTTTCGGCGGCGGCCGCGCGGCTTCCGAAGGAAATGCCAGGCGCTCAAGCCCA**TGTACA**GCATGATGAAC
R97G	AAGCGCCTGAC**GCTGAGC**GAGATCTACCAGTTCCTGCAGAGCCGCTTCCCCTTCTTCCGGGGCTCCTACCAGGGCTGGAAGAACTCCGTG**G**GCCACAACCTCTCGCTCAACGAGTGCTTCATCAAGCTACCCAAGGGCCTTGGGCGGCCCGGCAAGGGCCACTACTGGACCATCGACCCGGCCAGCGAGTTCATGTTCGAGGAGGGCTCCTTTCGGCGGCGGCCGCGCGGCTTCCGAAGGAAATGCCAGGCGCTCAAGCCCA**TGTACA**GCATGATGAAC
R97H	AAGCGCCTGAC**GCTGAGC**GAGATCTACCAGTTCCTGCAGAGCCGCTTCCCCTTCTTCCGGGGCTCCTACCAGGGCTGGAAGAACTCCGTGC**A**CCACAACCTCTCGCTCAACGAGTGCTTCATCAAGCTACCCAAGGGCCTTGGGCGGCCCGGCAAGGGCCACTACTGGACCATCGACCCGGCCAGCGAGTTCATGTTCGAGGAGGGCTCCTTTCGGCGGCGGCCGCGCGGCTTCCGAAGGAAATGCCAGGCGCTCAAGCCCA**TGTACA**GCATGATGAAC
H98Q	AAGCGCCTGAC**GCTGAGC**GAGATCTACCAGTTCCTGCAGAGCCGCTTCCCCTTCTTCCGGGGCTCCTACCAGGGCTGGAAGAACTCCGTGCGCCA**A**AACCTCTCGCTCAACGAGTGCTTCATCAAGCTACCCAAGGGCCTTGGGCGGCCCGGCAAGGGCCACTACTGGACCATCGACCCGGCCAGCGAGTTCATGTTCGAGGAGGGCTCCTTTCGGCGGCGGCCGCGCGGCTTCCGAAGGAAATGCCAGGCGCTCAAGCCCA**TGTACA**GCATGATGAAC
S101L	AAGCGCCTGAC**GCTGAGC**GAGATCTACCAGTTCCTGCAGAGCCGCTTCCCCTTCTTCCGGGGCTCCTACCAGGGCTGGAAGAACTCCGTGCGCCACAACCTCT**T**GCTCAACGAGTGCTTCATCAAGCTACCCAAGGGCCTTGGGCGGCCCGGCAAGGGCCACTACTGGACCATCGACCCGGCCAGCGAGTTCATGTTCGAGGAGGGCTCCTTTCGGCGGCGGCCGCGCGGCTTCCGAAGGAAATGCCAGGCGCTCAAGCCCA**TGTACA**GCATGATGAAC

Missense mutations are bolded and underlined, and the Blp1 and BsrGI recognition sites are bolded

pJ3M-MST1 (Addgene plasmid: #12203), pJ3MST1 K59R (Addgene plasmid: #12204), pJ3M-MST2 (Addgene plasmid: #12205) and pJ3M-MST2 K56R (Addgene plasmid: #12206) were gifts from Jonathan Chernoff, MD, PhD [22]. MST1 and MST1 K59R were cloned from the pJ3M plasmid into the pcDNA3.1 + plasmid by restriction with HindIII and EcoRI. To clone MST2 and MST2 K56R into pcDNA3.1 + , pJ3M plasmids were digested with SalI and EcoRI, and the pcDNA3.1 + plasmid was digested with HindIII and EcoRI. Blunt ends were created with Klenow, and MST1 and MST2 K56R inserts were ligated into the pcDNA3.1 + vector.

Constructs for luciferase assays were generated by cloning 5 repeats of the RTAAACA binding motifs or scrambled motifs separated by 10 nucleotides into the pGL4.10 [luc2] vector (Promega; E6651). DNA inserts were ordered as single-stranded oligonucleotides (IDTs) and annealed to make double-stranded DNA inserts, which are listed in Table 4. To each primer, a restriction site was added, 5’ for AccI and 3’ for XhoI, to clone the binding motif into the pGL4.10 [luc2] vector.

**Table 4:** sequences of Primers used for cloning the FOXF1 binding motifs into the pGL4.10[luc2] vector

Primer	Sequence 5′-> 3′
FOXO3 FW	gtaccAT**ATAAACA**CCACTAGAAT**ATAAACA**CCACTAGAAT**ATAAACA**CCACTAGAAT**ATAAACA**CCACTAGAAT**ATAAACA**CCA
FOXO3 RV	tcgaGG**TGTTTAT**ATTCTAGTGG**TGTTTAT**ATTCTAGTGG**TGTTTAT**ATTCTAGTGG**TGTTTAT**ATTCTAGTGG**TGTTTAT**ATG
FOXO3 scrambled FW	gtaccAT**AACAAAT**CCACTAGAAT**AACAAAT**CCACTAGAAT**AACAAAT**CCACTAGAAT**AACAAAT**CCACTAGAAT**AACAAAT**CCA
FOXO3 scrambled RV	tcgaTGG**ATTTGTT**ATTCTAGTGG**ATTTGTT**ATTCTAGTGG**ATTTGTT**ATTCTAGTGG**ATTTGTT**ATTCTAGTGG**ATTTGTT**ATG
CCNE2 FW	gtaccAT**GTAAACA**CCACTAGAAT**GTAAACA**CCACTAGAAT**GTAAACA**CCACTAGAAT**GTAAACA**CCACTAGAAT**GTAAACA**CCA
CCNE2 RV	tcgaTG**GTGTTTAC**ATTCTAGTGG**TGTTTAC**ATTCTAGTGG**TGTTTAC**ATTCTAGTGG**TGTTTAC** ATTCTAGTGG**TGTTTAC**ATG
CCNE2 scrambled FW	gtaccAT**CAAGAAT**CCACTAGAAT**CAAGAAT**CCACTAGAAT**CAAGAAT**CCACTAGAAT**CAAGAAT**CCACTAGAAT**CAAGAAT**CCA
CCNE2 scrambled RV	tcgaTGG**ATTCTTG**ATTCTAGTGG**ATTCTTG**ATTCTAGTGG**ATTCTTG**ATTCTAG TGG**ATTCTTG** ATTCTAGTGG **ATTCTTG**ATG

Binding motifs are in bold, and restriction sites are noncapitalized

### Immunofluorescence staining

Transfected HeLa cells on coverslips were fixed 48 h post-transfection with 4% paraformaldehyde for 10 min at room temperature (RT), permeabilized with 0.1% Triton-X/PBS for 10 min at RT and incubated for 20 min in blocking buffer (3% BSA in 0.05% Triton-X/PBS). Primary antibodies against FLAG (1:250, F7425 Sigma) were diluted in blocking buffer and incubated O.N. at 4 °C. The cells were subsequently washed and incubated for 1 h with fluorophore-conjugated secondary antibodies at RT. Subsequently, the cells were incubated with DAPI (1:2000, 564907; Becton and Dickinson) for 10 min at RT, and the coverslips were mounted using Mowiol 4–88 (81381 Sigma). The samples were imaged using a Leica TCS SP5 confocal laser scanning microscope and analysed using Fiji software.

### Protein extraction, SDS‒PAGE and phos-tag western blotting

For SDS‒PAGE, protein extracts of HEK293T cells were made as previously described [23], except for phosphorylation experiments, where RIPA buffer (150 mM NaCl, 1% NP-40, 0.5% Na-DOC, 0.1% SDS, 50 mM Tris pH 7.5) with complete EDTA-free protease inhibitor (CEF) (Roche) and PhosStop (Sigma) were used as protein extraction buffers for SDS‒PAGE. Proteins were separated by gel electrophoresis using a 4–12% ExpressPlus™ PAGE gel (GenScript) and transferred onto a PVDF membrane (Immobilon).

Phos-tag western blotting was performed according to the phos-tag product (AAL-107 M, Wako) manual protocol of Wako using the MnCl_2_ method with some modifications. Briefly, standard protein extracts of HEK293T cells were made in RIPA buffer (150 mM NaCl, 1% NP-40, 0.5% Na-DOC, 0.1% SDS, 50 mM Tris, pH 7.5) supplemented with complete EDTA-free protease inhibitor (CEF) (Roche) and PhosStop (Sigma). For phosphatase treatment, PhosStop was excluded from the protein extraction buffer, and the protein extract was incubated with rSAP (NEB) for 1 h at 37 °C. Proteins were separated by gel electrophoresis on a 10% running gel with 100 µM phos-tag using Tris–glycine buffer and transferred onto a PVDF membrane (Immobilon).

PVDF membranes were blocked using 3% BSA in 0.05% Tween in TBS and labelled with primary antibodies against FOXF1 (R&D, AF4798), MYC-tag (Abcam, ab9106), FLAG-tag (Sigma, F7425), HA-tag (Santa Cruz, sc-805), MST1 (Abcam, ab51134), MST2 (Abcam, ab52641) or cofilin (Abcam, ab42824) followed by HRP-conjugated secondary antibody labelling. Blots were developed using Pierce™ ECL Western Blotting Substrate (Thermo Fisher Scientific) and imaged on an Amersham Imager 600 (GE Healthcare).

### Co-immunoprecipitation experiments

Whole-cell lysates were made from transfected cells as described previously [23]. Co-immunoprecipitation experiments with WT expression constructs were performed with protein G agarose beads as previously described [23] or with Dynabeads™ Protein G Immunoprecipitation Kit (Invitrogen, 10007d) according to the manufacturer’s instructions for experiments with WT and kinase-dead expression constructs. Immunoprecipitation was performed with antibodies against MYC-tag (Roche, 1668149), FLAG-tag (Sigma, F1804) and HA-tag (Santa Cruz, sc-7392).

### Chromatin immunoprecipitation (ChIP) and motif analysis

A total of 20 × 10^6^ hECFCs were crosslinked in 1% formaldehyde, which was quenched with 0.125 M glycine. The cells were pelleted by centrifugation and snap-frozen for storage or sonicated. The cells were lysed in SDS lysis buffer (50 mM Tris, pH 8; 10 mM EDTA; 1% SDS; 1 × CEF), and the chromatin was sonicated for 15 cycles (30 s on, 30 s off per cycle) using a Bioruptor Pico (Diagenode). Chromatin was diluted with ChIP dilution buffer (167 mM NaCl, 16.7 mM Tris–HCl (pH 8), 1.1% Triton-X, 1.2 mM EDTA, 0.01% SDS with 1 × CEF). BSA-blocked protein G agarose beads (Millipore) were incubated O.N. at 4 °C with antibodies (FOXF1 AF4798 R&D or goat IgG R&D) and added to diluted chromatin for 3 h at 4 °C under gentle rotation. The beads were subsequently washed with low-salt immune buffer (150 mM NaCl, 20 mM Tris–HCl (pH 8), 2 mM EDTA, 1% Triton X, 0.1% SDS), high-salt immune buffer (500 mM NaCl, 20 mM Tris–HCl (pH 8), 2 mM EDTA, 1% Triton X, 0.1% SDS), LiCl immune complex buffer (250 mM LiCl, 10 mM Tris–HCl (pH 8), 1 mM EDTA, 0.5% NP-40, 0.5% Na-DOC) and TE buffer (10 mM Tris–HCl (pH 8), 1 mM EDTA). Bound chromatin was eluted with elution buffer (300 mM NaCl, 10 mM Tris–HCl (pH 8), 5 mM EDTA, 0.5% SDS) supplemented with 0.29 mg/ml proteinase K and incubated O.N. at 65 °C to decrosslink the chromatin. DNA was purified using a Qiagen MinElute column, and DNA libraries were prepared using the ThruPLEX DNA sample preparation protocol from Takara Bio and sequenced on an Illumina HiSeq2500 sequencer. Adapter sequences were removed from the sequence reads. These were aligned were aligned to the human GRCh38 reference genome using the HISAT2 aligner [24]. After alignment, duplicate reads were filtered and peaks were called with the MACS2 peak caller [25]. Sequence coverage over the genome was determined. For motif analysis, only peak sequences in promoter regions defined as 1 kb from transcription start sites were used, and motifs were identified with MEME-ChIP [26, 27].

### CUT&TAG

CUT&TAG experiments were performed with hECFCs according to a previously published protocol from the Henikoff laboratory, version 3 [28]. In brief, hECFCs were cultured until confluency, and a total of 11 × 10^6^ cells were harvested in 10% FCS/PBS and counted. Cells were isolated using Concanavalin A-coated (BP531-3 ml, Sanbio) beads. Primary antibodies against FOXF1 (AF4798, R&D) or the IgG control (AB-108-C, R&D) were incubated overnight at 4 °C, and the membranes were incubated with secondary antibodies for 1 h at room temperature. pA-Tn5 adapter complexes (C01070001, Diagenode) were bound at room temperature for 1 h, and tagmentation was performed for 1 h at 37 °C. DNA was extracted using the chloroform extraction method, and PCR was performed using NEBNext HiFi 2 × PCR master mix (M0541S) and indexed i5 and indexed i7 primers (Table 5). DNA was purified after PCR using SPRI paramagnetic beads (Agentcourt AMPure XP, A63880) and checked using a High DNA Sensitivity DNA assay on an Agilent Bioanalyzer 2100. Each sample was analyzed in duplicate, and DNA libraries and subsequent filtering and peak calling were performed as described above.

**Table 5:** CUT&TAG PCR primers

Name	Sequence 5′-> 3′
i5 v2_Ad1.1_TAGATCGC	AATGATACGGCGACCACCGAGATCTACACTAGATCGCTCGTCGGCAGCGTCAGATGTGTAT
I7 v2_Ad2.1_TAAGGCGA	CAAGCAGAAGACGGCATACGAGATTCGCCTTAGTCTCGTGGGCTCGGAGATGTG

### Electrophoretic mobility shift assay

EMSA assays were performed using the LightShift™ Chemiluminescent EMSA Kit (20,148 Thermo Scientific) according to the manufacturer’s instructions. Protein extracts were made as described for western blotting in Carin buffer. Briefly, all the samples were subjected to binding reactions and incubated for 20 min at RT. For binding reactions, biotin-labelled or nonlabelled DNA probes were used, and the sequences are listed in Table 6. Probes were ordered as single-stranded oligonucleotides and annealed to produce double-stranded DNA probes. After the binding reaction, the samples were loaded onto a native polyacrylamide gel with a ratio of 29:1 of acrylamide:bisacrylamide. First, 1 ml of 10 × Tris/borate/EDTA (TBE) buffer (900 mM Tris, 900 mM boric acid, 20 mM EDTA, pH 8.3), 2 ml of 40% acrylamide, 1.35 ml of N,N’-methylenebisacrylamide 2%, 200 µl of 10% ammonium persulfate and 20 µl of N,N,N,N-tetramethylenediamine were added to 15.43 ml of ultrapure water, and the mixture was run for 45 min at 100 V until ¾ of the gel. DNA was transferred to a Hybond XL nylon membrane (GE Healthcare) for 30 min at 380 mA and crosslinked to the membrane using a transilluminator equipped with 312 nm bulbs for 10 min. The membrane was blocked using blocking buffer included in the supplier’s kit and incubated with streptavidin–horseradish peroxidase conjugate. After the membrane was washed, the substrate equilibration and substrate working solution from the kit were added, and the membrane was imaged on an Amersham Imager 600 (GE Healthcare).

**Table 6:** Primer sequences for the EMSA DNA probes

Name	Sequence 5′-> 3′
CCNE2_GTAAACA_F	CTGCAGAATGTAAACACCACTCAGC
CCNE2_GTAAACA_R	GCTGAGTGGTGTTTACATTCTGCAG
CCNE2_BIO-GTAAACA_F	CTGCAGAATGTAAACACCACTCAGC
CCNE2_BIO-GTAAACA_R	GCTGAGTGGTGTTTACATTCTGCAG
Scrambled_GTAAACA_F	CTGCAGAATCAAGAATCCACTCAGC
Scrambled_GTAAACA_R	GCTGAGTGGATTCTTGATTCTGCAG
Scrambled_BIO-GTAAACA_F	CTGCAGAATCAAGAATCCACTCAGC
Scrambled_BIO-GTAAACA_R	GCTGAGTGGATTCTTGATTCTGCAG
FOXO3_ATAAACA_F	GAGCGAAACATAAACAAACGCACGC
FOXO3_ATAAACA_R	GCGTGCGTTTGTTTATGTTTCGCTC
FOXO3_BIO-ATAAACA_F	GAGCGAAACATAAACAAACGCACGC
FOXO3_BIO-ATAAACA_F	GCGTGCGTTTGTTTATGTTTCGCTC
Scrambled_ATAAACA_F	GAGCGAAACAACAAATAACGCACGC
Scrambled_ATAAACA_R	GCGTGCGTTATTTGTTGTTTCGCTC
Scrambled_BIO-ATAAACA_F	GAGCGAAACAACAAATAACGCACGC
Scrambled_BIO-ATAAACA_R	GCGTGCGTTATTTGTTGTTTCGCTC

### Luciferase assays

A luciferase assay was performed as previously described [29] with several modifications. HeLa cells were transiently transfected with Lipofectamine 3000 (Thermo Fisher, L3000001). Each transfection consisted of 50 ng of pcDNA3 expression plasmid, 50 ng of pGL4.10[luc2] reporter plasmid and 10 ng of TK-Renilla plasmid (Promega; E2241). Luciferase activity was measured 24 h after transfection using the Dual-Luciferase Reporter Assay System (Promega, E1910), and luminescence was measured with a VICTOR X4 plate reader. Each sample was normalized for transfection efficiency. An increase or decrease in luciferase activity was determined by normalizing luciferase activity to that of the scrambled motif control.

## Results

### FOXF1 missense mutations are mainly located in the FOXF1 DNA binding domain

**Fig. 1:**
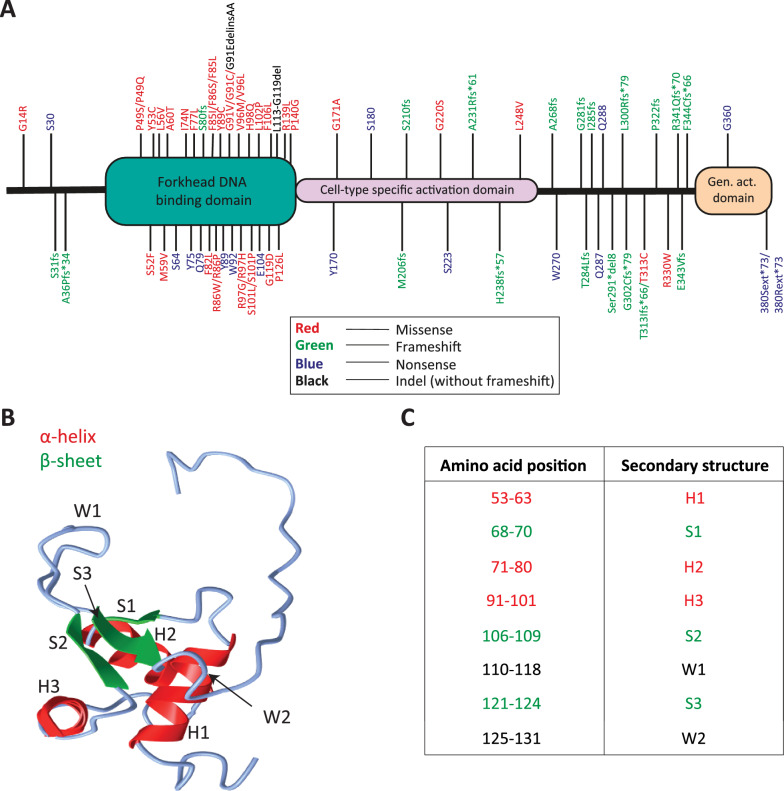
Distribution of *FOXF1* ACD/MPV patient mutations in the different FOXF1 protein domains and predicted 3D model of the FOXF1 DNA binding domain. **A** Genomic ACD/MPV patient mutations in the coding region of the *FOXF1* gene were translated to their corresponding amino acids to make an overview of the localization of the mutations in the FOXF1 protein. Forkhead DNA binding domain, cell-type specific activation domain and general activation domain (Gen. act. Domain) are shown in green, pink and orange, respectively. The locations of FOXF1 mutations are shown with lines and details of the mutations are shown next to them. Mutations were categorized in missense mutations (red), frameshifts (green), nonsense mutations (blue), insertion/deletions without a frameshift (Indel, black) and deletions (orange). **B** Predicted 3D model of the DNA binding domain of FOXF1, which consists of 2 wings (W), 3 α-helices (H) and 3 β-sheets (S). **C** Table of predicted secondary structures in the FOXF1 protein and their corresponding amino acid position in the FOXF1 protein

To study the effect of ACD/MPV patient-derived mutations on the molecular function of FOXF1, we first mapped known point mutations in the coding region of the *FOXF1* gene (Fig. 1A). Genomic *FOXF1* mutations were retrieved from the Leiden Open Variation database (LOVD) (https://databases.lovd.nl/shared/variants/FOXF1/unique) and recent publications that reported *FOXF1* mutations in ACD/MPV patients [17, 30–32]. The localization of the corresponding amino acid changes is graphically positioned on a linear representation of the FOXF1 protein (Fig. 1A). A relatively high number of frameshift mutations were located upstream of the general activation domain, resulting in a truncated, nonfunctional protein due to the loss of this domain. Interestingly, more than half of the identified mutations are located in the forkhead DNA-binding domain, and the majority are missense mutations (Fig. 1A). These results suggest that the mutations in the DNA binding domain affect the ability of FOXF1 to bind to its DNA recognition motif, which affects proper FOXF1 function.

### The G91-S101 region is frequently mutated

Next, a three-dimensional model of the forkhead DNA binding domain was generated. The DNA binding domain is encoded by amino acids 44–148 and was modelled using the resolved structures of human FOXK2, FOXO1, FOXO3, and FOXO4 as templates to predict the localization of the mutations in a three-dimensional structure [20]. The predicted model shows the typical winged helix domain consisting of two wings (W1 and W2) encoded by amino acids 110–118 and 125–131, respectively (Fig. 1B, C). Furthermore, there were three α-helices (H1, H2 and H3) encoded by amino acids 53–63, 71–80 and 91–101 and three β-sheets (S1, S2 and S3) encoded by amino acids 68–70, 106–109 and 121–124, respectively (Fig. 1B, C). We also compared our model with a 3D model predicted by AlphaFold, an artificial intelligence system that predicts a protein 3D structure based on its protein sequence [33, 34]. The α-helix H3 was differed by 1 amino acid position (92–102 instead of 91–101), and W2 was completely missing in the AlphaFold model (Figure S1). The other structural protein domains were comparable between the two models. Another paper that reported a model of the FOXF1 forkhead DNA binding domain suggested that the α-helix H3 is important for DNA interaction based on a generated 3D model [17]. Therefore, we focused on eighth missense mutations that are located in the α-helix H3, from now on, called the G91-S101 region (Fig. 1B, C; Table 7). The L56V mutation is located closely to the S52 residue, which may also play an important role in the interaction of FOXF1 with the DNA helix [17]. Additionally, three different missense mutations were identified for F85 (Fig. 1A). We previously generated induced pluripotent stem (iPS) cells from two patients with confirmed ACD/MPV and harboring either the L56V or F85I mutation [31]. For these reasons, both the L56V and F85I mutations were included, resulting in a total of 10 ACD/MPV-associated *FOXF1* mutations in this study (Table 7).

**Table 7:** ACD/MPV patient mutations in FOXF1 included in this study for molecular analysis

Mutation	Base change	Amino acid change
L56V	c.166C > G	p.Leu56Val
F85I	c.253T > A	p.Phe85Ile
G91V	c.272G > T	p.Gly91Val
V96L	c.286G > T	p.Val96Leu
V96M	c.286G > A	p.Val96Met
R97G	c.289C > G	p.Arg97Gly
R97H	c.290G > A	p.Arg97His
H98Q	c.294C > A	p.His98Gln
S101L	c.302C > T	p.Ser101Leu
S101P	c.301T > C	p.Ser101Pro

### FOXF1 mutants show diminished or no binding to DNA

**Fig. 2:**
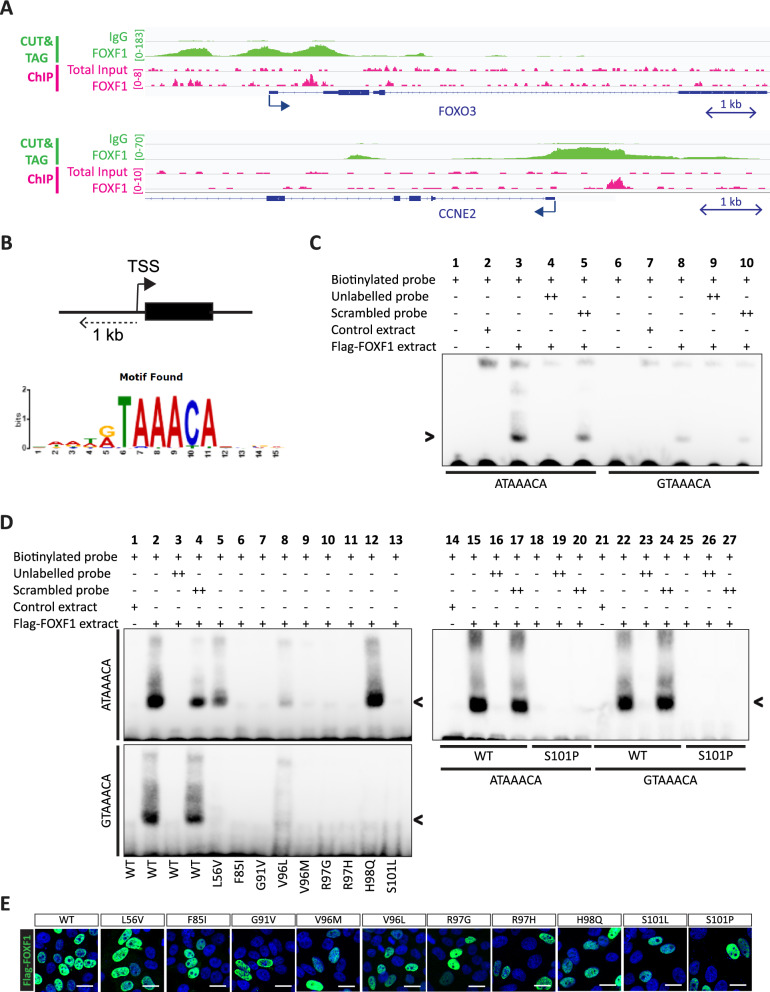
FLAG-tagged FOXF1 mutants have no or diminished binding to the FOXF1 binding motif. **A** FOXF1 ChIP-seq (pink) and CUT&TAG (green) analysis was performed in ECFCs and identified FOXF1 binding sites near transcriptional start sites of FOXO3 and CCNE2. Arrows indicate direction of transcription at transcriptional start site. **B** Motif analysis of FOXF1 ChIP-seq data in ECFCs identified a RTAAACA binding motif in promoter regions of FOXF1 target genes. ChIP-seq peaks located in 1 kb from transcription start site (TSS) were used for anaylsis as shown in the schematic. **C** EMSA-assay shows that WT FLAG-FOXF1 binds to the FOXO3 probe encoding the ATAAACA binding motif (A-motif) and to the CCNE2 probe containing the GTAAACA binding motif (G-motif). N = 3. **D** EMSA-assay shows that FLAG-L56V, FLAG-V96L and FLAG-H98Q have diminished binding to the FOXO3 probe. Other FOXF1 mutants show no binding. N = 3. WT: wild-type. Arrowhead indicates a band shift. **E** FLAG-tagged FOXF1 mutant proteins have no altered intracellular localization. Wild-type of mutant FLAG-tagged FOXF1 proteins were overexpressed in HepG2 cells and stained with immunofluorescence for the FLAG-tag to show intracellular localization of FOXF1. Scale bar = 20 µm

To investigate the effects of patient-derived mutations on the function of the FOXF1 protein, we introduced the selected mutations into a FLAG epitope-tagged FOXF1 expression plasmid. Since most of these mutations are located in the putative DNA binding domain, we first examined the effects of the mutations on the DNA binding properties of the FOXF1 protein. FOXF1 ChIP-seq of human endothelial colony forming cells (hECFCS) followed by motif analysis revealed a RTAAACA FOXF1 binding motif present in the promoter regions of FOX1 bound protein-coding genes (Fig. 2A, B). ChIP-seq analysis revealed that the promoter region of the *FOXO3* gene contained the ATAAACA sequence, while the promoter region of the *CCNE2* gene contained the GTAAACA sequence. Both genes were amongst the top most enriched binding regions in ChIP-seq analysis and additionally, results were validated by CUT&TAG analysis, which confirmed the presence of functional FOXF1 binding sites in the promoter regions of both genes (Fig. 2A). An electrophoretic mobility shift assay (EMSA) was performed to examine the in vitro binding capacities of the normal and mutant FOXF1 proteins to the GTAAACA (G-motif) and ATAAACA (A-motif) binding motifs. DNA probes were designed that contained either the G-motif or A-motif, while probes with a mutated core binding motif (“scrambled”) served as negative controls. HEK293T cells were transfected with FLAG epitope tagged wild-type (WT) or mutant FOXF1 expression constructs, and protein extracts were used to perform in vitro binding assays. Mock-transfected protein extracts (“control extract”) did not show probe retention, or a shift (Fig. 2C, lane 2), whereas extracts with the WT FOXF1 showed efficient binding to the A-motif, as shown by a clear shift caused by the slower migration of the probe-protein complex (Fig. 2C, lane 3, arrowhead). The binding of the WT-FOXF1 to the biotinylated probe was efficiently competed by adding excess unlabelled probe, as shown by the loss of the shifted band (Fig. 2C, lane 4), but was not competed by the addition of excess unlabelled scrambled probe (Fig. 2C, lane 5). FLAG-FOXF1 also bound to the G-motif, although with reduced efficiency as compared to the binding to the A-motif (Fig. 2C, lanes 8–10). We also compared the binding properties of normal, wild-type (WT) FOXF1 tagged at the N-terminus (FLAG-FOXF1) or C-terminus (FOXF1-FLAG) (Figure S2A). Surprisingly, FOXF1-FLAG did not bind to the A-motif (Figure S2A), indicating that the C-terminal FLAG-tag interfered with DNA binding. These data show that WT FOXF1 physically and specifically binds to the A- and G-motifs as identified by FOXF1 ChIP-seq analysis.

Next, we analysed the binding properties of the different FOXF1 mutant proteins using the A-motif and G-motif. This revealed that the L56V, V96L and H98Q mutants had reduced binding capacity (Fig. 2D, lanes 5, 8 and 12) to the A-motif, whereas the other mutants completely lost the DNA binding property (Fig. 2D, lanes 6, 7, 9–1 and 13). Western blot analysis revealed that this reduced or absent binding was not caused by differences in protein expression between FOXF1 mutants and WT FOXF1 (Figure S2B,C) or altered protein localization (Fig. 2E). Interestingly, the H98Q mutant FOXF1 seemed to have a greater binding affinity for the A-motif than the wild-type FOXF1 (Fig. 2D, lane 12). This binding capacity, as well as the affinity of the other mutants, was completely abolished for the G-motif (Fig. 2D). This suggests that the nucleotide change from A to G 5’ of the core binding sequence has a major impact on the DNA binding of FOXF1 mutant proteins. One exception is the V96L mutant, which equally well binds to both motifs. Collectively, these data show that ACD/MPV patient-specific mutations in the FOXF1 G91-S101 region, as well as two mutations outside this region, affect the binding of the FOXF1 protein to its identified core binding motifs albeit to different extents. In addition, these results indicate that α-helix H3 of FOXF1 is import for binding to the DNA-helix.

### FOXF1 mutants exhibit aberrant transcriptional activity

**Fig. 3:**
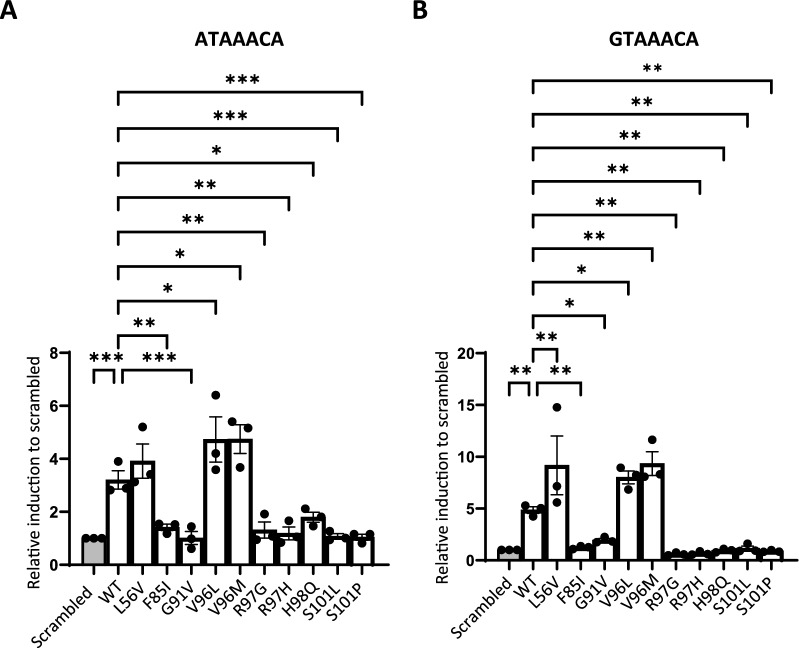
FOXF1 mutants show aberrant transcriptional activity. Luciferase assay to show transcriptional activity of FLAG-tagged WT FOXF1 or FOXF1 mutants to the binding motif containing minimal promoter of (A) *FOXO3* (ATAAACA) gene (A-motif) and (B) *CCNE2* (GTAAACA) gene (G-motif). One-way ANOVA (n = 3; *p < 0.05, **p < 0.01, ***p < 0.001 compared to scrambled). WT: wild-type

Having established that some FOXF1 mutants are still able to bind to the core DNA binding motif in vitro, we next investigated whether the binding of these mutants would also lead to transcriptional activation using in vitro luciferase assays. Therefore, the A- or G-motif was first cloned into a luciferase reporter construct, after which HEK293T cells were transiently co-transfected with a FLAG-FOXF1 expression construct and the luciferase reporter. The WT FOXF1 showed comparable transcriptional activity for both the A- and G-motif (Fig. 3). As expected, all FOXF1 mutants that did not bind to the core motifs in the in vitro EMSA assay, also lacked transcriptional activity (Fig. 3). Compared to WT FOXF1, the L56V and V96L mutants showed increased transcriptional activity with the G-motif (Fig. 3B), and the V96L mutant also showed increased transcriptional activity with the A-motif (Fig. 3A). Interestingly, the V96M mutant showed increased transcriptional activity with both motifs (Fig. 3), although it did show poor binding capacity (Fig. 2C). Surprisingly, despite strong binding to the binding motifs, the H98Q mutant showed no transcriptional activity (Fig. 3), which was not caused by a reduced expression (Figure S3). These results show that the majority of mutant FOXF1 proteins that still bind to the core DNA motif have aberrant transcriptional activity compared to that of WT FOXF1.

### FOXF1 phosphorylation is altered in FOXF1 mutants

Transcription factors frequently form protein complexes to control gene expression through regulating DNA binding capacity or transcriptional activity, or may form complexes to regulate other molecular processes [35]. To investigate putative FOXF1-interacting proteins in human lung endothelial colony-forming cells (hECFCs), FOXF1 complexes were enriched via immunoprecipitation and associating proteins were identified by mass-spectrometry analysis (manuscript in preparation). Interestingly, a total of three kinases, serine/threonine-protein kinases 3 and 4 (STK3/4, also named Macrophage Stimulating 1/2 (MST1/2)), aurora kinase B (AURKB) and WNK lysine deficient protein kinase 1 (WNK1), were identified as potential FOXF1-interacting proteins. To investigate whether these three kinases were capable of phosphorylating FOXF1, we first used prediction tool GPS5.0 to predict whether these kinases were able to phosphorylate FOXF1 and if so, which serine or threonine in the FOXF1 protein would be phosphorylated [36]. Selection of MST1/2, AURKB and WNK1 with a low threshold analysis resulted in 3 potential serines, S95, S101 and S136, as potential target sites for phosphorylation by MST1/2 (

**Fig. 4:**
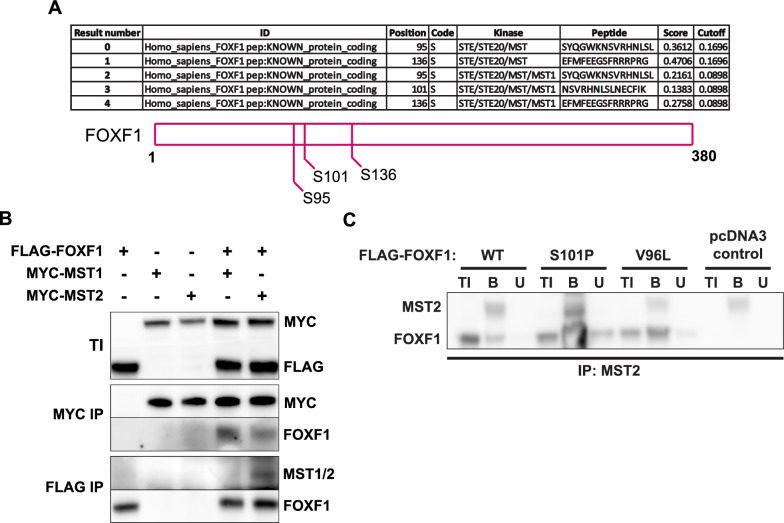
FOXF1 interacts with MST1/2. **A** Sites in the FOXF1 protein that are predicted to be phosphorylated by MST1/2 using GPS5.0. Below the table is a schematic representation of the FOXF1 protein and potential phosphorylated sites. **B** Western blot results of co-immunoprecipitation of transfected epitope-tagged FOXF1 and MST1/2 in HEK293T cells. Proteins were immunoprecipitated with antibodies against the indicated epitopes (MYC IP or FLAG IP) and Westerrn blots were labelled with antibodies against MYC, FLAG, FOXF1 or MST1/2. **C** Western blot results of co-immunoprecipitation experiments with transfected epitope-tagged FOXF1 and endogenous MST2. Immunoprecipitation was performed with antibodies against MST2 and blots were labelled with antibodies for MST2 and FOXF1. MST2 expression in TI was below detection level. TI: total input, IP: immunoprecipitation. U: unbound

Figure 4A). Interestingly, two point mutations of S101 have previously been identified in ACD/MPV patients, which could affect phosphorylation of the FOXF1 protein (Table 7) [3, 32]. Based on these predictions, we decided to validate the interaction between FOXF1 and MST1/2. Therefore, we transiently expressed the WT FLAG-FOXF1 and either a MYC-tagged MST1 or MYC-tagged MST2 in HEK293T cells followed by immunoprecipitation and western blot analysis Fig. 4B). Precipitation of the MST1 or MST2 with the MYC-epitope antibody showed co-precipitation of FOXF1 (Fig. 4B, MYC-IP), and precipitation of FOXF1 with the FLAG-epitope antibody showed association of MST2 (Fig. 4B, FLAG-IP). This confirmed the physical interaction between FOXF1 and both MST1 and MST2 (Fig. 4B). We also evaluated the interaction of a FOXF1 mutant that retained and lost DNA-binding capacity and transcriptional activity, V96L and S101P mutants respectively, with MST2. Both FOXF1 mutants were still able to interact with MST2 (Fig. 4C), indicating that these mutations do not affect protein–protein interaction.

**Fig. 5:**
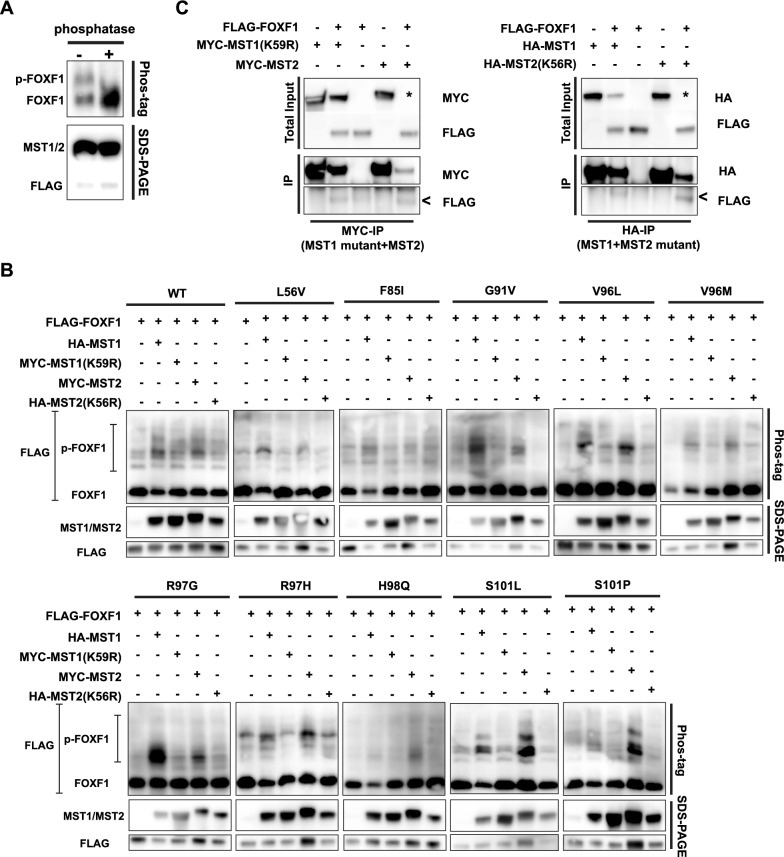
Phosphorylation is affected in FOXF1 mutant proteins containing ACD/MPV-related mutations. **A** Results of phos-tag and SDS-PAGE western blot of transfected epitope-tagged FOXF1 and MST1 in HEK293T cells. Extracts were treated with phosphatase to demonstrate specificity of the phosphorylated FOXF1 band in the phos-tag results. Blots were labelled with antibodies against FLAG-tag or MST1/2 antibody mix. **B** Phos-tag (top) and SDS-page western blots (middle and bottom) results of co-transfected epitope-tagged FOXF1 wild-type (WT) or FOXF1 mutants with MST1/2 WT or catalytically inactive MST1/2 in HEK293T cells. Phos-tag blot was labeled with the anti-FLAG antibody to detect tagged FOXF1 proteins, and SDS-PAGE western blots were labelled with antibodies against MST1/2 (middle) or FLAG-tag (bottom) to evaluate protein levels. **C** Co-immunoprecipitation of extracts of HEK cells co-transfected with WT FLAG-FOXF1 and WT MST1/2 or kinase-dead MST1(K59R) and MST2 (K56R) proteins. The top shows the total input of the immunoprecipitated protein extracts. After immunoprecipitation with antibodies against the MYC (left)-or HA-tag (right), co-precipitated FLAG-FOXF1 is detected (bottom). Succesful precipitation was confirmed by labeling the blots with anti-MYC or anti-HA antibody (middle). MST2 wild-type and kinase-dead mutant could not be detected upon overexpression with FLAG-FOXF1 in total input samples, indicated by *. Nevertheless, MST2 was purified after immunoprecipitation, indicating that it was expressed, but below detection level in total input fraction

To investigate the potential phosphorylation of FOXF1 and the FOXF1 mutants by MST1/2 kinase activity, we first investigated whether phosphorylated FOXF1 could be detected. Therefore, the phos-tag system was used, which is a based on a phosphate affinity acrylamide gel that separates proteins by their level of phosphorylation [37]. HEK293T cells were transiently transfected with MYC-MST1 and FLAG-FOXF1 expression constructs and a protein extract was subsequently separated on a phos-tag acrylamide gel to visualize phosphorylated proteins (Fig. 5A). Phosphorylated and non-phosphorylated FOXF1 were clearly detected as visualized by two separate bands (5A, left lane). The phosphorylated, upper band disappeared upon rSAP phosphatase treatment of the extract, while the intensity of the lower band increased, indicating an enrichment of unphosphorylated FOXF1 (Fig. 5A, right lane). Regular Western blotting was performed to confirm protein expression (Figura 5A). These results show that both phosphorylated and non-phosphorylated FOXF1 can be detected simultaneously and that FOXF1 is potentially phosphorylated by MST1.

Next, we wanted to validate whether FOXF1 phosphorylation was a result of MST1/2 kinase activity. Therefore, FLAG-tagged WT FOXF1 or FOXF1 mutants were co-transfected either with WT HA-tagged MST1 or mutant MYC-tagged MST1(K59R), or WT MYC-tagged MST2 or mutant HA-tagged MST2(K56R) into HEK293T cell, of which mutant MST1/2 lack kinase activity. After transfection and protein extraction, phos-tag western blotting was performed to study the effect on phosphorylation of FOXF1 (Fig. 5B). Although only traces of endogenous MST1 or MST2 were detected in the single transfected WT FOXF1 sample with standard western blotting, a clear phosphorylated FOXF1 (p-FOXF1) could be detected using the phos-tag gel (Fig. 5B). Upon co-expression with WT MST1, a greater proportion of p-FOXF1 was detected, while phosphorylation upon co-expression with MST1(K59R), the kinase-dead mutant, was comparable to the WT FOXF1 single transfected sample (Fig. 5B). The effect of MST2 WT and the kinase-dead mutant MST2 on FOXF1 phosphorylation followed the same trend as that of MST1 and the kinase-dead mutant MST1 (Fig. 5B). Like WT MST1/2, the kinase-dead MST mutants were able to physically interact with FOXF1 (Fig. 5C), thus excluding the possibility that mutant MST1/2 could not phosphorylate FOXF1 because these proteins could not associate with FOXF1. Thus, these results show that MST1 and MST2 are able to directly phosphorylate FOXF1.

Next, WT and kinase-dead mutant MST1/2 with FOXF1 mutants were co-expressed to study the phosphorylation of FOXF1 mutants by MST1/2 (Fig. 5B). The most striking differences with the WT FOXF1 were observed with the G91V, R97G and S101 mutants. Interestingly, more p-FOXF1 was observed in the S101P mutant co-expressed with WT MST2, while MST1 co-expression resulted in a comparable amount of p-FOXF1 to that of WT FOXF1 (Fig. 5B). In the S101L mutant, p-FOXF1 phosphorylation was increased with both MST1 and MST2, with a ratio of phosphorylated:unphosphorylated FOXF1 of approximately 1:1. In addition, p-FOXF1 was more prominent and pronounced in the S101 mutants than in the in WT FOXF1 (Fig. 5B). Increased phosphorylation and an approximate ratio of phosphorylated:unphosphorylated FOXF1 of 1:1 were also observed for the R97G and G91V mutants with MST1, similar to the S101L mutant, whereas p-FOXF1 in the H98Q mutant seemed to be reduced (Fig. 5B). In the other mutants, the effects of MST1 and MST2 on phosphorylation appeared comparable to those of WT FOXF1 (Fig. 5B). These results indicate that mutations in the DNA binding domain of the FOXF1 protein may lead to conformational changes that interfere with phosphorylation by MST1 and MST2. Taken together, these results show that MST1 and MST2 can phosphorylate WT FOXF1 and that phosphorylation of some mutant FOXF1 proteins is altered in ACD/MPV.

## Discussion

In the present study, we found that missense mutations in the coding region of the *FOXF1* gene identified in patients with the lethal condition ACD/MPV were enriched in the G91-S101 region of the winged helix DNA binding domain of the protein, indicating that amino acid changes in this conserved region likely affects FOXF1 function. We focused on identifying why these mutations impaired FOXF1 function by analyzing the effect of ten individual point mutations in this domain of the FOXF1 protein on its DNA binding capacity, its role as transcriptional activator and its phosphorylation status. At the time of this study, the G91C mutation had not yet been reported [38], and therefore only the G91V mutation was included in this study.

The binding capacity of individual FOXF1 mutants was analyzed by an in vitro DNA binding assay, which revealed that the majority of the mutants exhibited either an altered or no binding to the two FOXF1 DNA binding motifs identified by in vitro chromatin immunoprecipitation assays. Interestingly, more FOXF1 mutants retained binding capacity to the FOXO3 based A-motif than to the CCNE2 based G-motif, where only the V96L mutant retained residual binding capacity. Although the two probes only differed in the first base of a 7-base binding motif, this minor difference had a significant impact on the binding of FOXF1 mutants. Remarkably, the in vitro transcription results of the luciferase assays showed that DNA binding not exclusively determines transcriptional activation. The FOXF1 L56V, V96L and H98Q mutants all retained DNA binding capacity, but the H98Q mutant lost transcriptional activity. In contrast, the L56V and V96L mutants showed increased transcriptional activity. These variations can be caused by the (in)capability of FOXF1 mutants to recruit additional factors that are important for transcriptional activation or by affecting gene expression indirectly through other proteins or protein complexes. The latter would provide an explanation for the result of the V96M mutant, which lost DNA binding in the EMSA but showed increased expression in the luciferase assay. Nevertheless, the corresponding results of the EMSA assays and luciferase assays support a loss of direct DNA binding capacity with the majority of ACD/MPV-associated missense mutants.

These results imply that each FOXF1 DNA binding domain mutant may have different effects on the regulation of target genes, depending on the DNA-binding motif present in the promoter region, which adds complexity to the development of ACD/MPV and may explain the phenotypic heterogeneity observed in patients. Linking the molecular relationship between missense mutations and transcriptional regulation of specific molecular pathways may reveal shared pathways that are affected at the basis of ACD/MPV, or identify different or mutation-specific pathways that contribute to phenotypic heterogeneity. Future molecular and functional studies should expand the FOXF1 mutant panel beyond the G91-S101 region and outside the DNA-binding domain to obtain more insight into this relationship. For example, according to the AlphaFold model (Figure S1) and a previously published study [17], the G119 amino acid is an interesting amino acid for studying protein‒DNA interactions as it is predicted to bind the DNA helix, and missense mutations and deletions have been identified in ACD/MPV patients at this particular position. In addition, the effect of mutant FOXF1 on DNA binding and target gene expression should be studied further in patient material or in vitro in cellular models that genetically carry each of the different FOXF1 mutations to validate our findings for clinical significance.

We could not find a relationship between the physicochemical properties of the amino acid substitutions and their effect on DNA binding. Usually, substitutions between chemically distant amino acids, also called radical replacements, have a more dramatic effect than substitutions between chemically close amino acids, also called conservative replacements. In our study, both conservative and radical replacement altered binding to the FOXF1 binding motif. FOXF1 mutants show altered binding to the A-motif present in the *FOXO3* promoter region. FOXO3 is a transcription factor that is involved in multiple cellular processes, and altered *FOXO3* levels have previously been shown to affect endothelial progenitor cell proliferation [39, 40]. Single-cell sequencing of ACD/MPV and control infant lungs also revealed reduced *FOXO3* expression in capillary endothelial cells in ACD/MPV [41]. These data suggest a potential role for dysregulated *FOXO3* expression in ACD/MPV, which may contribute to decreased endothelial cell proliferation resulting in reduced capillary density in the lungs of patients with ACD/MPV.

At the protein and molecular levels, the FOXF1 S52F mutation has been studied more extensively before. The FOXF1 S52F mutation disrupts the interaction with STAT3, resulting in decreased expression of STAT3 target genes [17]. Furthermore, the S52F mutation resulted in altered cellular localization of the FOXF1 protein [17]. We also checked for our investigated mutations but did not observe altered localization compared to that of the FOXF1 WT protein (Fig. 2E), showing that altered gene regulation is not caused by inaccessibility of the protein to the DNA in this study, but by altered binding capacity to DNA.

Multiple phosphorylated FOXF1 bands were observed using the phos-tag system, indicating that the FOXF1 protein was phosphorylated at multiple sites. In several FOXF1 mutants, phosphorylation was affected, and especially in the S101 FOXF1 mutants, combined phosphorylation by MST1 and MST2 was increased, which was in contrast with our expectations. Phosphorylation mainly occurs at the site of three amino acid side chains, namely, serine, threonine and tyrosine [42]. MST1 and MST2 are serine-threonine kinases, so mutation of serine at this position to anything other than threonine was expected to result in reduced FOXF1 phosphorylation. It is possible that mutation in the DNA binding regions of FOXF1 increases the accessibility of other potential phosphorylation sites, leading to increased phosphorylation. Posttranslational modifications, such as phosphorylation, are important cellular regulatory mechanisms, and it has been reported that almost 5% of disease-causing amino acid substitutions may affect protein function through posttranslational modifications [43]. Recently, ubiquitination by the E3 ubiquitin ligase HECTD1 was shown to be important for degradation of FOXF1 [44]. Interference with the interaction between these two proteins by the novel compound TanFe stabilized the FOXF1 protein, and treatment of pregnant mice with TanFe prevented postnatal mortality in Foxf1^±^ mice [44]. This shows that posttranslational modifications of FOXF1 may be important for regulating FOXF1 function in ACD/MPV.

## Conclusion

In summary, this study revealed that missense mutations in the DNA binding domain of the *FOXF1* gene affect binding of FOXF1 to its binding motifs as well as its transcriptional activity and posttranscriptional modification status. Future studies should focus on the specific molecular pathways affected by these mutations and the effect of posttranslational modifications on FOXF1 protein function.

## Supplementary Information

Supplementary Material 1: Figure S1: Predicted 3D model of the FOXF1 DNA-binding domain. A) 3D structure of the DNA binding domain of FOXF1 as predicted by AlphaFold [33, 34] showing the same three α-helices (H) and three β-sheets (S) as in our model (Figure 1) and as previously published [17]. One wing (W1) is comparable to our model, but the structure of the second wing is different. G119 and S52 in green are predicted to bind the DNA helix together with α–helix H3. B) Secondary structure with their corresponding amino acid position, according to AlphaFold.

Supplementary Material 2: Figure S2: FLAG-tagged FOXF1 binding is influenced by the location of the FLAG-tag, but not FOXF1 expression level. A) EMSA-assay with either N-terminal FLAG-tagged FOXF1 (FLAG-FOXF1, left) or C-terminal FLAG-tagged FOXF1 (FOXF1-FLAG, right) with the A-motif. Only FLAG-FOXF1 binds to the A-motif encoding the ATAAACA binding motif. Arrowhead indicates a shift. B,C) Western blot of FLAG-FOXF1 protein extracts used in the EMSA-assays in Figure *3*2. Blots are labelled with antibodies against the FLAG-tag and cofilin as loading control. Missense mutations are shown with the WT amino acid first represented by their 1-letter code, followed by the amino acid position in the coding region of the FOXF1 protein and the amino acid change that results from the genomic mutation.

Supplementary Material 3: Figure S3: Western blot of extracts used for luciferase assays. Extracts used for the luciferase assays were analyzed for protein expression of the transfected FLAG-tagged FOXF1 proteins. Proteins were separated by electrophoresis followed by SDS-PAGE and western blots were labelled with antibodies against the FLAG-tag, and cofilin is used as loading control. Missense mutations are shown with the WT amino acid first represented by their 1-letter code, followed by the amino acid position in the coding region of the FOXF1 protein and the amino acid change that results from the genomic mutation below the blots. Binding motifs are indicated on the left of the blot.

## Data Availability

The datasets used and/or analysed during the current study are available from the corresponding author upon reasonable request.

## Abbreviations

ACD/MPV: Alveolar capillary dysplasia with misalignment of pulmonary veins
AURKB: Aurora kinase B
ECFC: Endothelial colony forming cells
EMSA: Electrophoretic mobility shift assay
FCS: Fetal calf serum
FOXF1: Forkhead box F1
iPS: Induced pluripotent stem
LOVD: Leiden Open Variation database
MST1: Macrophage Stimulating 1
MST2: Macrophage Stimulating 2
O.N.: Overnight
P/S: Penicillin–streptomycin
RT: Room temperature
WNK1: WNK lysine deficient protein kinase 1
