# Supplementary figures and images for: The molecular consequences of FOXF1 missense mutations associated with alveolar capillary dysplasia with misalignment of pulmonary veins
Source: J Biomed Sci. 2024 Nov 4;31:100. doi: 10.1186/s12929-024-01088-5 (PMC11536904; doi:10.1186/s12929-024-01088-5)

**A**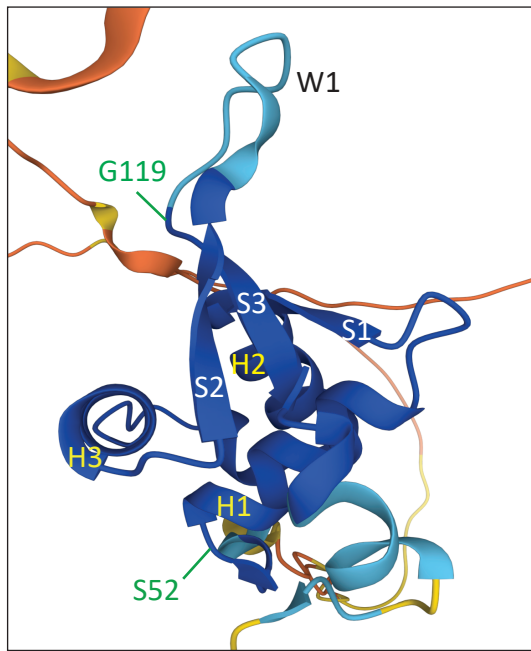**B**

| Amino acid position | Secondary structure |
|---------------------|---------------------|
| 53-62               | H1                  |
| 68-70               | S1                  |
| 71-81               | H2                  |
| 92-102              | H3                  |
| 106-109             | S2                  |
| 110-118             | W1                  |
| 121-124             | S3                  |

Supplement: Supplementary file 1 — Supplementary Material 1: Figure S1: Predicted 3D model of the FOXF1 DNA-binding domain. A) 3D structure of the DNA binding domain of FOXF1 as predicted by AlphaFold [33, 34] showing the same three α-helices (H) and three β-sheets (S) as in our model (Figure 1) and as previously published [17]. One wing (W1) is comparable to our model, but the structure of the second wing is different. G119 and S52 in green are predicted to bind the DNA helix together with α–helix H3. B) Secondary structure with their corresponding amino acid position, according to AlphaFold. [file 12929_2024_1088_MOESM1_ESM.pdf]

**A**

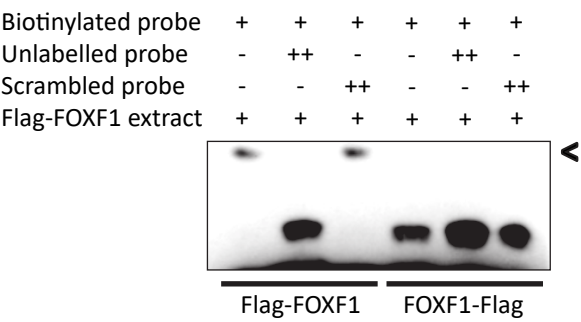

**B**

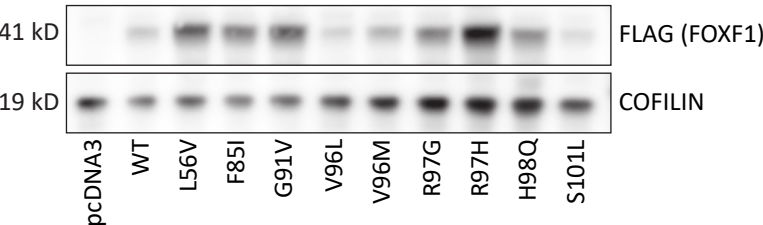

**C**

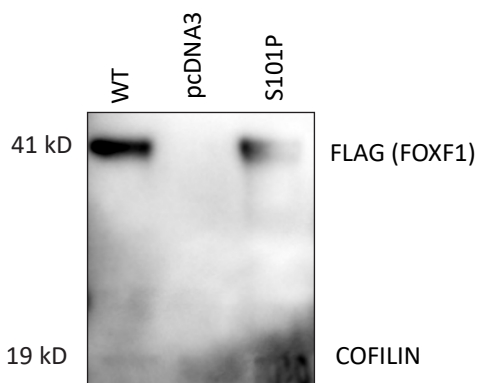

Supplement: Supplementary file 2 — Supplementary Material 2: Figure S2: FLAG-tagged FOXF1 binding is influenced by the location of the FLAG-tag, but not FOXF1 expression level. A) EMSA-assay with either N-terminal FLAG-tagged FOXF1 (FLAG-FOXF1, left) or C-terminal FLAG-tagged FOXF1 (FOXF1-FLAG, right) with the A-motif. Only FLAG-FOXF1 binds to the A-motif encoding the ATAAACA binding motif. Arrowhead indicates a shift. B,C) Western blot of FLAG-FOXF1 protein extracts used in the EMSA-assays in Figure 32. Blots are labelled with antibodies against the FLAG-tag and cofilin as loading control. Missense mutations are shown with the WT amino acid first represented by their 1-letter code, followed by the amino acid position in the coding region of the FOXF1 protein and the amino acid change that results from the genomic mutation. [file 12929_2024_1088_MOESM2_ESM.pdf]

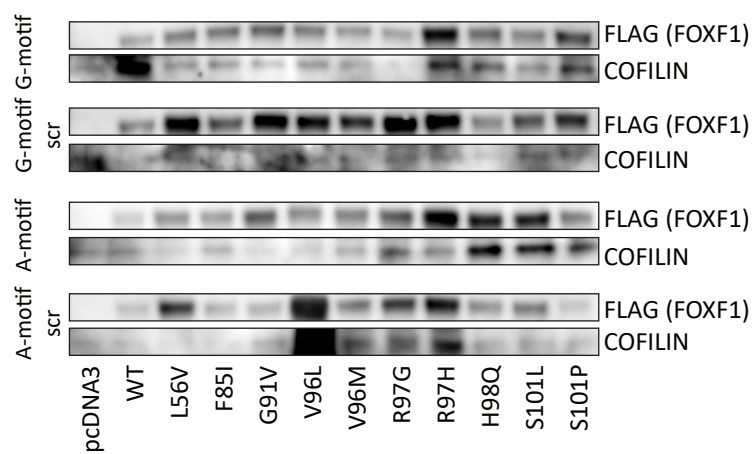

Supplement: Supplementary file 3 — Supplementary Material 3: Figure S3: Western blot of extracts used for luciferase assays. Extracts used for the luciferase assays were analyzed for protein expression of the transfected FLAG-tagged FOXF1 proteins. Proteins were separated by electrophoresis followed by SDS-PAGE and western blots were labelled with antibodies against the FLAG-tag, and cofilin is used as loading control. Missense mutations are shown with the WT amino acid first represented by their 1-letter code, followed by the amino acid position in the coding region of the FOXF1 protein and the amino acid change that results from the genomic mutation below the blots. Binding motifs are indicated on the left of the blot. [file 12929_2024_1088_MOESM3_ESM.pdf]
